# Modulation of hearing function following the downgrading or upgrading of endolymphatic hydrops in Meniere’s disease patients with endolymphatic duct blockage

**DOI:** 10.1371/journal.pone.0240315

**Published:** 2020-10-30

**Authors:** Anquan Peng, Junjiao Hu, Qin Wang, Wenqi Jiang, Wei Liu, Zhiwen Zhang, Chao Huang, Yichao Chen

**Affiliations:** 1 Department of Otolaryngology, The Second Xiangya Hospital, Central South University, Changsha, Hunan, China; 2 Department of Radiology, The Second Xiangya Hospital, Central South University, Changsha, Hunan, China; Universidade Federal de Sao Paulo/Escola Paulista de Medicina (Unifesp/epm), BRAZIL

## Abstract

The present study was to investigate the dynamics of endolymphatic hydrops (EH) and hearing function, and explore whether the hearing loss is caused by EH alone and whether the hearing function can be modulated by changes in the EH. The extent of EH visualized by gadolinium (Gd)-enhanced inner ear magnetic resonance imaging, hearing thresholds and the summating potential/action potential ratio (-SP/AP ratio) of electrocochleography (ECochG) were recorded prior to and following surgery in 22 patients with intractable Meniere’s disease (MD) who underwent endolymphatic duct blockage (EDB). The difference value of the hearing threshold and -SP/AP ratio was significantly positively correlated with the difference value of the endolymph to vestibule-volume ratio (EVVR) and grading of cochlear hydrops between prior to and following surgery. Among patients with a decreased EVVR, the average hearing threshold and -SP/AP ratio was significantly decreased following surgery, as compared to that prior to surgery. Six out of seven patients with a hearing improvement (≥10-dB decline) and 4/5 patients with a negative conversion of EcochG showed downgrading of their hydrops in the cochlea and/or vestibule. By contrast, among patients with an increased EVVR, the average hearing threshold and -SP/AP ratio tended to increase following EDB, as compared with that prior to surgery. One out of two patients with a hearing deterioration (≥10-dB elevation) showed upgrading of her hydrops in both cochlea and vestibule. The present results showed the downgrading of cochlear and/or vestibular hydrops accompanied by the downregulation of the hearing threshold and -SP/AP ratio of EcochG, as well as the upgrading of cochlear and/or vestibular hydrops that tended to upregulate the hearing threshold and -SP/AP ratio of EcochG; this suggested that hearing loss is likely to be caused by hydrops and that the impaired hearing function be modulated by changes in the hydrops.

## Introduction

Meniere’s disease (MD) is a well-recognized but inadequately understood condition associated with the classic triad of episodic vertigo, low-tone fluctuating sensorineural hearing loss and tinnitus. Numerous studies have confirmed that virtually all cases of ‘‘true” MD present with endolymphatic hydrops (EH) in the inner ear [1–4]. However, certain studies have proposed that the presence of EH in MD may be a mere epiphenomenon, with no direct causal relationship to the disease [5]. The association between EH and Meniere’s symptoms has been questioned [6]. Yet, in the last decade, in vivo visualization of EH by MRI has provided compelling evidence linking EH to clinical symptoms of MD [7–10]. A mounting body of evidence from radiologically-proven hydrops has identified a strong correlation between the extent of hearing loss and that of hydrops, particularly in the low tone range [11–15]. However, the way in which the hydrops affects hearing function is not fully understood.

In the present study, the dynamics of EH were evaluated using gadolinium (Gd)-enhanced inner ear MRI, changes in hearing thresholds and summating potential/action potential ratio (-SP/AP ratio) of electrocochleography (ECochG) in a group of patients who had undergone endolymphatic duct blockage (EDB) for the treatment of MD; the aim was to clarify whether or not the change of hydrops is likely to modulate the hearing function and -SP/AP ratio, which would verify a cause-effect relationship between hydrops and auditory physiology.

## Materials and methods

### Patients

This study was conducted at a large tertiary referral center. Between January 2018 and December 2018, a total of 2,160 patients with vertigo or dizziness were referred to our otology-led vertigo clinic. According to the diagnostic criteria for MD jointly formulated by the Classification Committee of the Bárány Society [16], 62 patients were diagnosed with unilateral definite MD, 8 patients with bilateral definite MD and 18 patients with unilateral probable MD. Out of these 88 patients with definite or probable MD, 39 with intractable MD were hospitalized and referred for 3T MRI of the temporal bone, which demonstrated EH. A total of 26 patients (12 females and 14 males; age range, 30–72 years; mean age, 51.2 years) with intractable MD and MRI-based visualization of unilateral EH underwent EDB for the treatment of MD. Intractable MD in patients was defined as recurrent vertigo/dizziness for ≥6 months, with a failure of systematic medical treatment and psychological management, including appropriate life guidance (i.e., reduction of stress, prevention of overworking and appropriate exercise), as well as oral therapy involving the administration of osmotic diuretic medicine, ameliorants of inner ear circulation, cyanocobalamin and traditional Chinese medicine.

The present study was approved by the Medical Ethics Committee of the Second Xiangya Hospital (certificate number: S452). All participants provided their written informed consent in accordance with the Declaration of Helsinki. Authors had access to information that could identify individual participants during or after data collection.

On the day of the examination, that is, immediately before contrast medium application and 1 day before magnetic resonance imaging (MRI), patients underwent pure tone audiometry (PTA) and extratympanic ECochG either prior to or following surgery. Following the confirmation of both cochlear and vestibular hydrops by Gd-enhanced MRI and once informed consent was obtained, the patients underwent surgery. From February 10 to March 30, 2020, a total of 22 patients were recalled for repeat PTA, ECochG and MRI scans examinations. A total of 4 patients were excluded from the study, due to lack of follow-up Gd-MRI scans, while 22 subjects (11 females and 11 males; age range, 30–65 years; mean age, 50.1 years) met the inclusion criteria.

Hearing function was measured by a pure-tone audiometer and was evaluated based on the four-tone average air-conductive hearing threshold formulated by (a+b+c+d)/4; a, b, c and d represent hearing levels at 0.25, 0.5, 1.0 and 2.0 kHz, respectively. Changes in the hearing levels were defined as worse (≥10-dB elevation), better (≥10-dB decline) or same (-10 dB< x <10 dB).

### ECochG

The silver-ball electrode for EcochG was placed on the upper wall of the deepest part of the ear canal near the eardrum, and extratympanic ECochG was recorded bilaterally by averaging 1,000 sweeps after alternating click stimuli (rate: 15/sec) with levels of 100 dB nHL. This test was considered positive at an -SP/AP of >0.40 at our hospital. For further analysis reasons, cases where EcochG traces were unobtainable were classed as negative.

### Gd-MRI

MRI was performed using a single-dose (0.2 ml/kg) intravenous administration of gadopentetate dimeglumine (Magnevist^®^, Bayer AG) 4 h before the MRI scan (IV-Gd), as well as intratympanic administration of 8-fold-diluted Gd (IT-Gd) in both ears or the ear undergoing surgery for the follow-up Gd-MRI, if patients were reluctant to receive intratympanic injections in their healthy ear, 24 h prior to the MRI scan. All scans were performed on a 3T MRI scanner (Magnetom Verio; Siemens AG) using a 12-channel head coil. Three-dimensional real inversion recovery (3D-real IR) sequence MRI images were collected, as previously described [7–9]. Briefly, the parameters for the 3D-real IR sequence were: Voxel size, 0.4x0.4x0.8 mm; scan time, 14 min; repetition time (TR), 9,000 msec, echo time (TE), 181 msec; inversion time (TI), 1,730 msec; slice thickness, 0.80 mm; field of view (FOV), 160x160 mm; matrix size, 3,300x918. The off-label use of IT-Gd-MRI was performed under informed consent.

### Image evaluation

**Fig 1:**
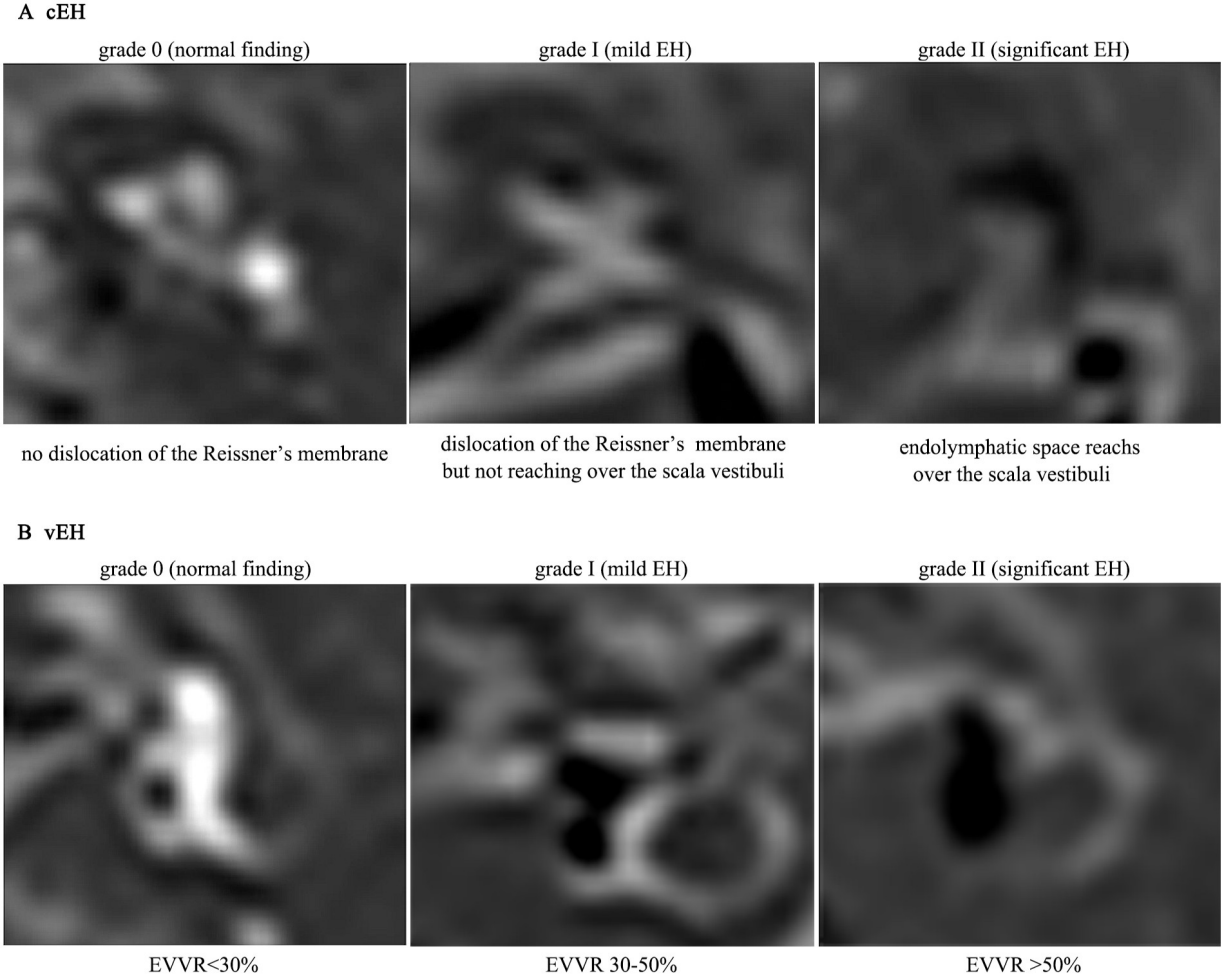
Criteria for EH-grading of the cochlea and vestibule in MRI performed 4 h after intravenous and 24 h after intratympanic administration of Gd-based contrast media (IV-Gd + IT-Gd MRI). (A) The criterion for cEH was the dislocation of the Reissner’s membrane. (B) The decisive factor for vEH is the EVVR. EH, endolymphatic hydrops; MRI, magnetic resonance imaging; Gd, gadolinium; IV-Gd, intravenous injection of gadopentetate dimeglumine; IT-Gd, intratympanic injection of gadopentetate dimeglumine; cEH, cochlear EH; vEH, vestibular EH; EVVR, endolymph to vestibule-volume.

**Fig 2:**
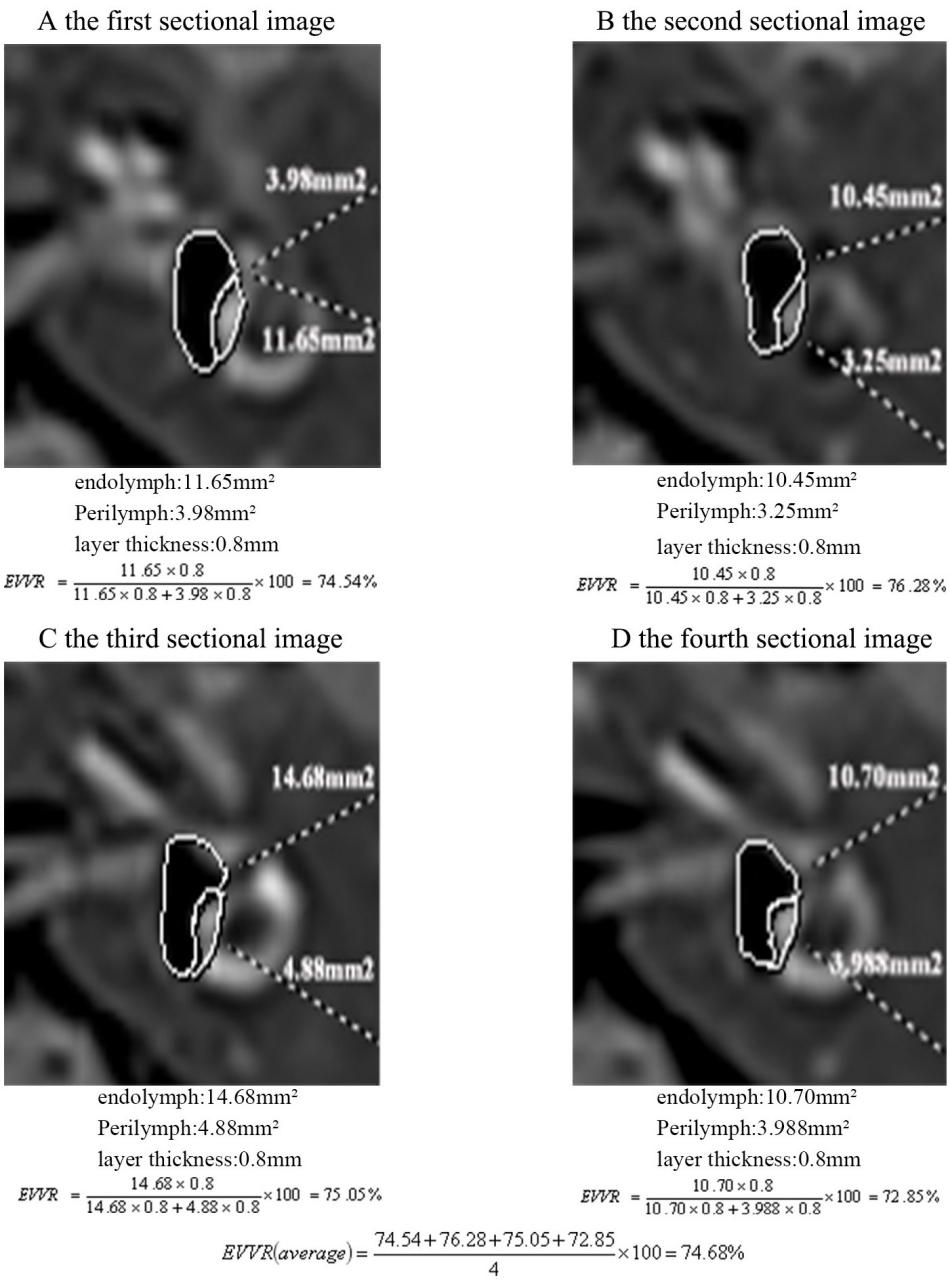
Assessment of the EVVR. For the EH-grading of the vEH, the overall EVVR was calculated by measuring the area of the xn and yn in every section plane showing the vestibule, and then using the average of those values. The volume of both endolymphatic and perilymphatic space was calculated by multiplying the measured x or y, by the uniform layer thickness of 0.8 mm (a). EVVR, endolymph to vestibule-volume; EH, endolymphatic hydrops; vEH, vestibular EH; xn, endolymph; yn, perilymph; x or y, surface area.

All the images obtained by MRI were evaluated by two experienced radiologists who were blinded to the diagnosis of all patients. Analysis of the boundary between the perilymphatic and endolymphatic spaces facilitated the detection of EH, as it was evidenced as an enlarged negative signal expanding into the positive signal of the perilymphatic space confined to the bony labyrinth using 3D-real IR after IT-Gd + IV-Gd. According to the criteria previously described by Nakashima et al and Wesseler et al [9,17], the degree of EH in the cochlea was assessed by visual comparison of the relative areas of the non-enhanced endolymphatic space vs. the contrast-enhanced perilymph space in the axial plane on the mid-mediolar level with regards to a possible dislocation of the Reissner’s membrane. The degree of cochlear hydrops was categorized as none (normal finding without EH), grade I (mild EH) or grade II (significant EH) (Fig 1A). An EH of the vestibule was determined by the volume-ratio of endolymphatic space to the total vestibule (endolymph to vestibule-volume ratio, EVVR) using the Syngo.via software package (VB20A; Siemens AG); None, <30% of the vestibular space was filled with endolymph; grade I, 30–50% of the vestibular space was filled with endolymph; grade II, >50% of the vestibular space was filled with endolymph (Fig 1B). According to the suggestion of Wesseler et al [17], this ratio was not estimated based on one section plane alone, but was measured separately in every plane showing the vestibule then using the average of those values calculated as the overall result. The number of planes varied between three and four. The volume of both endolymphatic and perilymphatic space was calculated by multiplying the measured surface area (x or y) by the uniform layer thickness of 0.8 mm (a). Thereby, the interpersonal variability in the assessment of an image was minimized. Fig 2 shows and exemplary illustration of this process.

Endolymphtovestibule-volumeratio=EVVR[%]

The enhancement of vestibular hydrops was defined as an EVVR increase of >10 points (%). The reduction of vestibular hydrops was defined as an EVVR decrease of >10 points (%). No change in the vestibular hydrops was defined as an EVVR change of ≤10 points (%). The complete reversal of vestibular hydrops was defined as an EVVR of <30 points (%).

### Surgical technique

**Fig 3:**
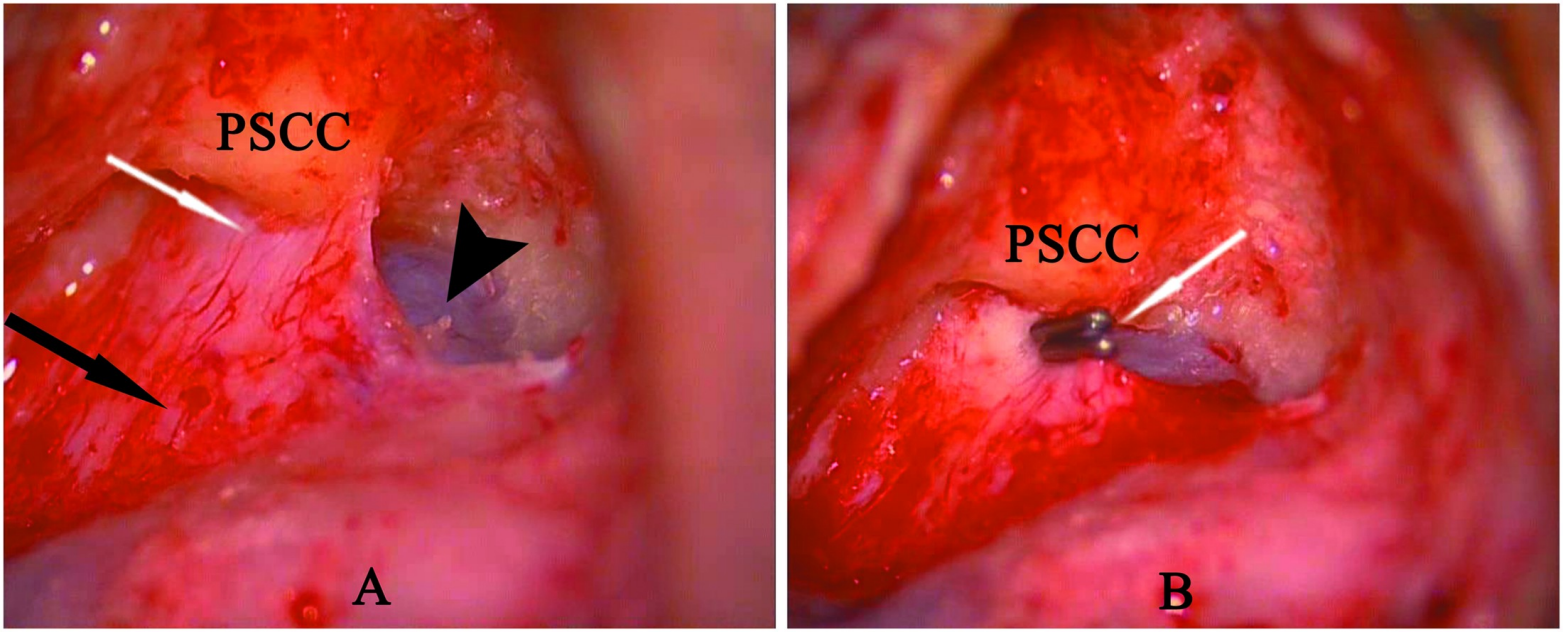
(A) Left ear mastoidectomy and endolymphatic sac dissection showing the intraosseous portion of sac (white arrow), extraosseous portion of sac (black arrow) and posterior fossa dura (arrow head). (B) Two titanium clips (white arrow) blocking the endolymphatic duct behind the posterior semicircular canal (PSCC).

The surgical procedure used for EDB was similar to that described previously [18]. The technical details were as follows: A simple mastoidectomy was performed, clearly exposing the endolymphatic sac in the area between the sigmoid sinus and the inferior margin of the posterior semicircular canal, including the rugose portion. Following the complete skeletonization and decompression of the sac, the bone of the vestibular aqueduct operculum and the posterior fossa dura were dissected from the retrolabyrinthine bone medial to the sac to identify, and to create a region for the insertion of the tips of the forceps to clip the proximal portion of the extraosseous endolymphatic sac. Finally, two small titanium clips were used to block the dissected endolymphatic duct by clipping using the ligating clip applier Small pieces of absorbable gelatin sponge soaked in high-concentration dexamethasone were placed on the surface of the sac (Fig 3). Postoperative wound management and postoperative care were similar to those used in other mastoid surgical operations.

### Statistical analysis

For statistical analysis, we used paired Student’s t-tests for two-group comparisons and Pearson correlations for evaluating the relationship. p values lower than .05 were considered significant. All data were statistically treated with SPSS version 26.0 (IBM Corp., Armonk, NY).

## Results

Table 1 shows the clinical profiles of all 22 patients prior to and following surgery, including the results of IV-Gd + IT-Gd MRI, audiometry and ECochG examination in the corresponding periods. Preoperatively, all 22 patients with typical and active unilateral MD showed cochlear and vestibular hydrops in the affected ear; no significantly dilated endolymphatic space could be documented in the asymptomatic contralateral ears. T2-weighted cisternography sequence was used to rule out vestibular schwannoma or endolymphatic sac tumor. No misregistration artifact from motion was noted in any image dataset of the ears. None of the subjects showed no or diagnostically insufficient inner ear contrast. Either prior to or following surgery, PTA was carried out in the whole cohort and EcochG in 95.5% (21/22), as EcochG traces were unobtainable in 1 patient (patient no. 19) with severe hearing loss.

**Table 1:** Clinical profiles in all 22 patients prior to and following surgery.

Case No.	Age/Sex/Side	Hearing(dB)			-SP/AP ratio			EVVR(%)			Cochlear hydrops			follow-up
		Pre	Post	D-value	Pre	Post	D-value	Pre	Post	D-value	Pre	Post	D-value	(Mon)
**1**	**65/M/L**	**58.8**	**55**	**-3.8**	**0.66**	**0.75**	**+0.09**	**42.60**	**46.82**	**+4.22**	**I**	**I**	**0**	**21**
**2**	**51/M/L**	**48.8**	**45**	**-3.8**	**1.0**	**0.85**	**-0.15**	**66.78**	**63.50**	**-3.28**	**I**	**I**	**0**	**21**
**3**	**37/M/L**	**55**	**57.5**	**+2.5**	**0.15**	**0.36**	**+0.21**	**56.42**	**59.55**	**+1.55**	**I**	**I**	**0**	**21**
**4**	**39/F/R**	**37.5**	**25**	**-12.5**	**0.60**	**0.16**	**-0.44**	**74.68**	**15.45**	**-59.23**	**I**	**N**	**-1**	**20**
**5**	**50/F/R**	**65**	**38.8**	**-26.2**	**1.61**	**0.22**	**-1.39**	**87.75**	**26.90**	**-60.85**	**II**	**I**	**-1**	**20**
**6**	**61/F/L**	**56.3**	**66.3**	**+10.0**	**0.45**	**0.55**	**+0.10**	**46.55**	**52.10**	**+5.55**	**I**	**I**	**0**	**19**
**7**	**47/F/R**	**46.3**	**35**	**-11.3**	**0.24**	**0.37**	**+0.13**	**53.65**	**51.50**	**-2.15**	**I**	**I**	**0**	**19**
**8**	**52/F/L**	**46.3**	**58.8**	**+12.5**	**0.94**	**0.78**	**-0.16**	**37.85**	**52.30**	**+14.45**	**I**	**II**	**+1**	**18**
**9**	**65/F/R**	**65**	**73.8**	**+8.8**	**1.0**	**1.45**	**+0.45**	**70.22**	**84.45**	**+14.23**	**II**	**II**	**0**	**18**
**10**	**46/M/R**	**55**	**36.3**	**-18.7**	**1.02**	**0.52**	**-0.50**	**82.65**	**47.45**	**-35.20**	**II**	**II**	**0**	**18**
**11**	**64/F/R**	**72.5**	**65**	**-7.5**	**0.57**	**0.72**	**+0.15**	**60.85**	**62.30**	**+1.45**	**II**	**II**	**0**	**17**
**12**	**30/F/L**	**68.8**	**33.8**	**-35.0**	**0.92**	**0.19**	**-0.73**	**49.28**	**10.35**	**-39.03**	**I**	**N**	**-1**	**17**
**13**	**48/M/R**	**56.3**	**58.8**	**+2.5**	**0.42**	**0.51**	**+0.09**	**54.55**	**46.25**	**-8.30**	**I**	**I**	**0**	**16**
**14**	**62/F/R**	**55**	**26.3**	**-28.7**	**0.95**	**0.22**	**-0.73**	**78.73**	**13.89**	**-64.84**	**II**	**I**	**-1**	**16**
**15**	**54/M/R**	**63.8**	**67.5**	**+3.7**	**0.32**	**0.38**	**+0.06**	**76.22**	**78.35**	**+2.13**	**II**	**II**	**0**	**15**
**16**	**48/F/L**	**52.5**	**33.8**	**-18.7**	**0.49**	**0.42**	**-0.07**	**83.50**	**47.28**	**-36.22**	**II**	**II**	**0**	**15**
**17**	**48/M/L**	**43.8**	**47.5**	**+3.7**	**0.63**	**0.80**	**+0.17**	**41.30**	**45.15**	**+3.85**	**I**	**I**	**0**	**15**
**18**	**52/M/L**	**60**	**56.3**	**-3.7**	**1.02**	**1.22**	**+0.20**	**66.82**	**64.25**	**-2.57**	**II**	**II**	**0**	**14**
**19**	**60/M/R**	**80**	**78.8**	**-1.2**	**absence**	**absence**		**58.18**	**52.75**	**-5.43**	**II**	**II**	**0**	**14**
**20**	**39/M/R**	**43.8**	**50**	**+6.2**	**0.37**	**0.62**	**+0.25**	**46.88**	**70.45**	**+23.57**	**I**	**II**	**+1**	**14**
**21**	**49/F/R**	**47.5**	**51.3**	**+3.8**	**0.30**	**0.28**	**-0.02**	**50.65**	**47.48**	**-3.17**	**I**	**I**	**0**	**13**
**22**	**36/F/L**	**36.3**	**35**	**-1.3**	**0.50**	**0.28**	**-0.22**	**38.15**	**41.35**	**+3.20**	**I**	**I**	**0**	**13**

Including the results of IV-Gd + IT-Gd MRI, audiometry and ECochG examination in the corresponding periods. D-value: Difference of value; EVVR: Endolymph to vestibule-volume ratio; F: Female; M: Male; Pre: Preoperative; Post: Postoperative; L: Left; R: Right; Mon: Month; N: No endolymphatic hydrops; I: Mild endolymphatic hydrops; II: Significant endolymphatic hydrops; 0: No change; +1: Upgrading; -1: Downgrading.

### Evaluation of EH on IT-Gd + IV-Gd MRI prior to and following surgery

According to MRI scans, the grade of the cochlear EH in 22 patients was subjectively rated as follows: Mild EH (I) in 13 ears and significant EH (II) in 9 ears prior to surgery, and no hydrops (N) in 2 ears, mild EH (I) in 11 ears and significant EH (II) in 9 ears following surgery. A total of 4 patients showed downgrading of cochlear hydrops and 1 patient showed upgrading (Table 1).

Based on the EVVR results, the vestibular EH in 22 patients was classified as follows: Mild EH (I) in 7 ears and significant EH (II) in 15 ears prior to surgery, and no hydrops (N) in 4 ears, mild EH (I) in 7 ears and significant EH (II) in 11 ears following surgery. A total of 6 patients showed downgrading of vestibular hydrops and 2 patients showed upgrading (Table 1). The average EVVR was 60.19±15.62 prior to and 49.09±19.25 following surgery in 22 patients, and it decreased significantly following EDB (P = 0.042). With regards to the difference value of the EVVR between prior to and following surgery, 12 patients showed a decreased EVVR value, and 10 patients showed an increased EVVR value. However, a reduction of vestibular hydrops was identified in 6 patients who showed an EVVR decrease of >10 points (-35.20 to -64.84%), and increased vestibular hydrops was detected in 3 patients who showed an EVVR increase of >10 points (14.23 to 23.57%). The other 13 patients showed no change in the vestibular hydrops, as their EVVR changed by ≤10 points (-8.30 to 5.50%). There was a significant positive correlation between the difference value of the EVVR and the grading of cochlear hydrops prior to and following surgery (r = 0.702, P = 0.000), which indicated that the change of hydrops in the vestibule and cochlea was synchronized.

**Fig 4:**
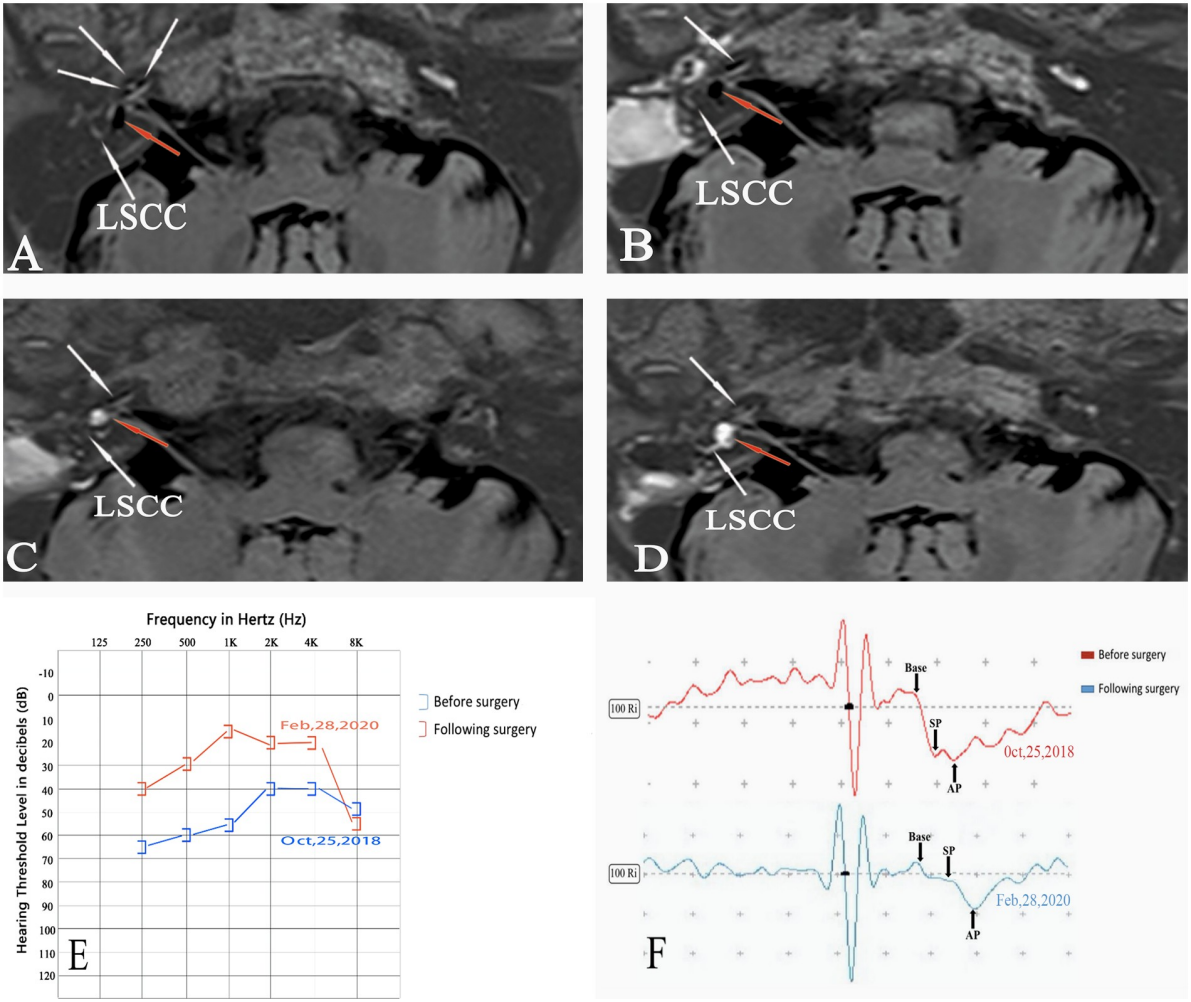
3D-real IR MRI scans of patient no. 14 (Table 1) with right MD. (A) prior to surgery, (B) 2 weeks, (C) 10 months and (D) 16 months after surgery, as well as changes of the hearing threshold and -SP/AP ratio of EcochG prior to and following surgery. (A) Axial IT-Gd + IV-Gd MRI scan of the right ear showing a significant EH in the vestibule (red arrow) and cochlea (white arrow) and no pathological finding in the left unaffected ear. (B) A significant EH in the vestibule (red arrow) and cochlea (white arrow) remained unchanged 2 weeks after surgery in the right ear. (C) Downgrading of vestibular EH from grade II to none (EVVR, 24.25%) and a remaining grade II EH in the cochlea 10 months after surgery. (D) Axial IT-Gd + IV-Gd MRI showing downgrading of EH from grade II to none (EVVR, 13.89%) in the vestibule (red arrow) and from grade II to I (white arrow) in the cochlea 16 months after surgery in the right ear in the same section plane as in parts A-C. Axial IV-Gd MRI scan of the left unaffected ear showing insufficient contrast in the basal turn of the cochlea. (E) Audiogram showing that the mean hearing threshold of air conduction (0.25, 0.5, 1.0 and 2.0 KHz) decreased from 55 dB before to 26.3 dB after surgery. (F) A negative conversion of EcochG was detected as the -SP/AP ratio decreased from 0.95 before to 0.22 (Table 1) after surgery. LSCC, lateral semicircular canal; 3D-real IR, three-dimensional real inversion recovery; MRI, magnetic resonance imaging; -SP/AP, summating potential/action potential ratio; Gd, gadolinium; EcochG, electrocochleography; EH, endolymphatic hydrops; EVVR, endolymph to vestibule-volume; MD, Meniere’s disease; IT-Gd, intratympanic injection of gadopentetate dimeglumine; IV-Gd, intravenous injection of gadopentetate dimeglumine.

**Fig 5:**
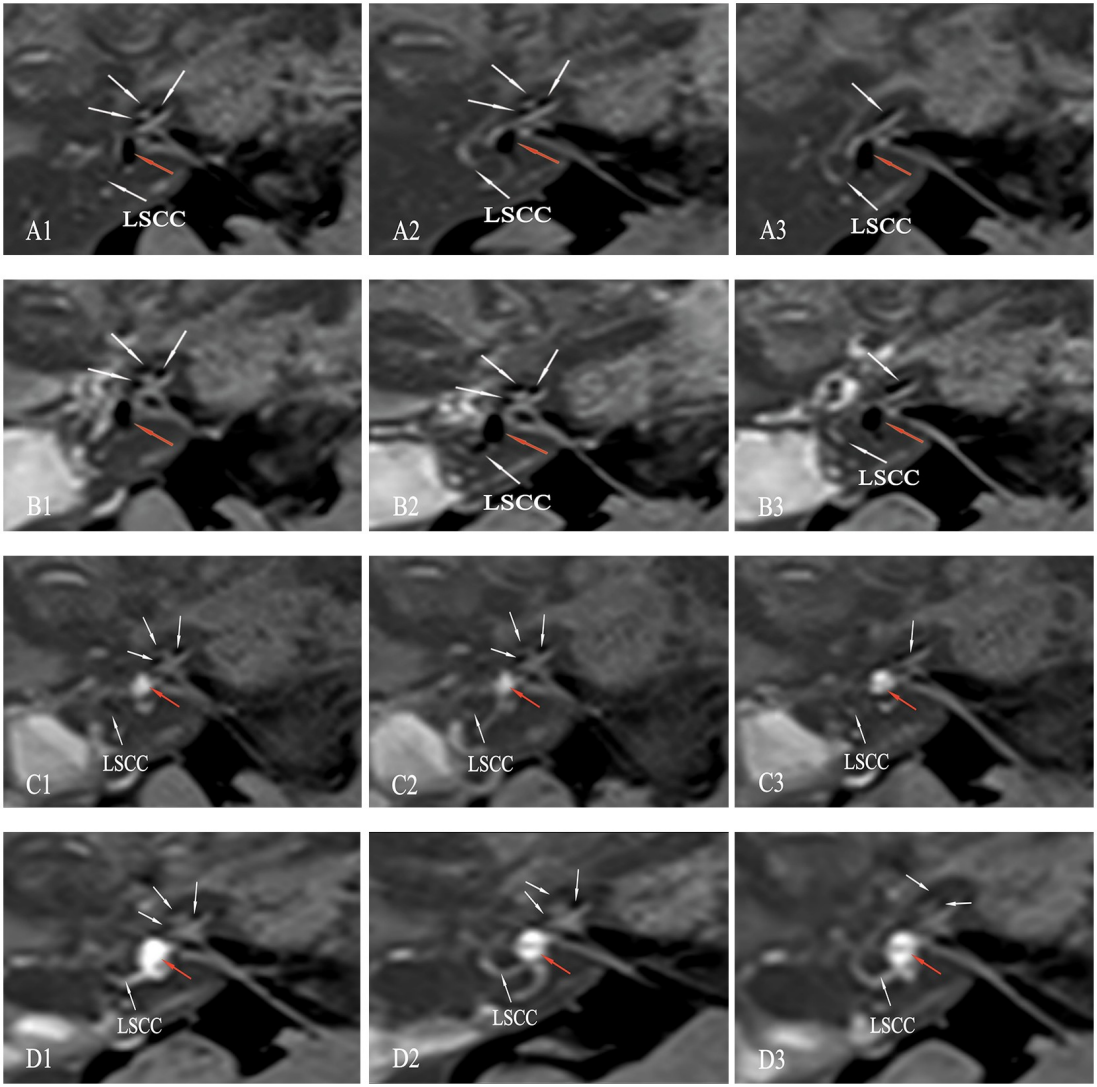
3D-real IR serial MRI scans of patient no. 14 (Table 1) with right MD. (A1-3) prior to surgery, and (B1-3) 2 weeks, (C1-3) 10 months and (D1-3) 16 months after surgery. (A1-3) Serial MRI scans showing a significant EH in the vestibule (red arrow) and cochlea (white arrow) before surgery. (B1-3) Unchanged EH in the vestibule (red arrow) and cochlea (white arrow) 2 weeks after surgery. (C1-3) Reversal of vestibular hydrops and remaining grade II EH in the cochlea 10 months after surgery. (D1-3) Serial MRI scans showing a disappearance of vestibular EH (red arrow) and the reduction of cochlear EH (white arrow) 16 months after surgery. LSCC, lateral semicircular canal; 3D-real IR, three-dimensional real inversion recovery; MRI, magnetic resonance imaging; EH, endolymphatic hydrops; MD, Meniere’s disease.

Figs 4 and 5 shows the dynamic changes of EH in patient no. 14, who underwent 4 Gd-MRI scans prior to surgery (October 26, 2018), and 2 weeks (November 8, 2018), 10 months (August 30, 2019) and 16 months (February 29, 2020) after surgery. Axial IT-Gd + IV-Gd MRI of the right ear showed a significant EH in the vestibule and cochlea prior to surgery (Figs 4A and 5A1-3), an unchanged hydrops 2 weeks after surgery (Figs 4B and 5B1-3), downgrading of vestibular EH from grade II to none (EVVR, 24.25%) and a remaining grade II EH in the cochlea 10 months after surgery (Figs 4C and 5C1-3), and downgrading of EH from grade II to none (EVVR, 13.89%) in the vestibule and from grade II to I in the cochlea 16 months after surgery (Figs 4D and 5D1-3).

### Modulation of the hearing threshold following dynamic changes in the hydrops

**Fig 6:**
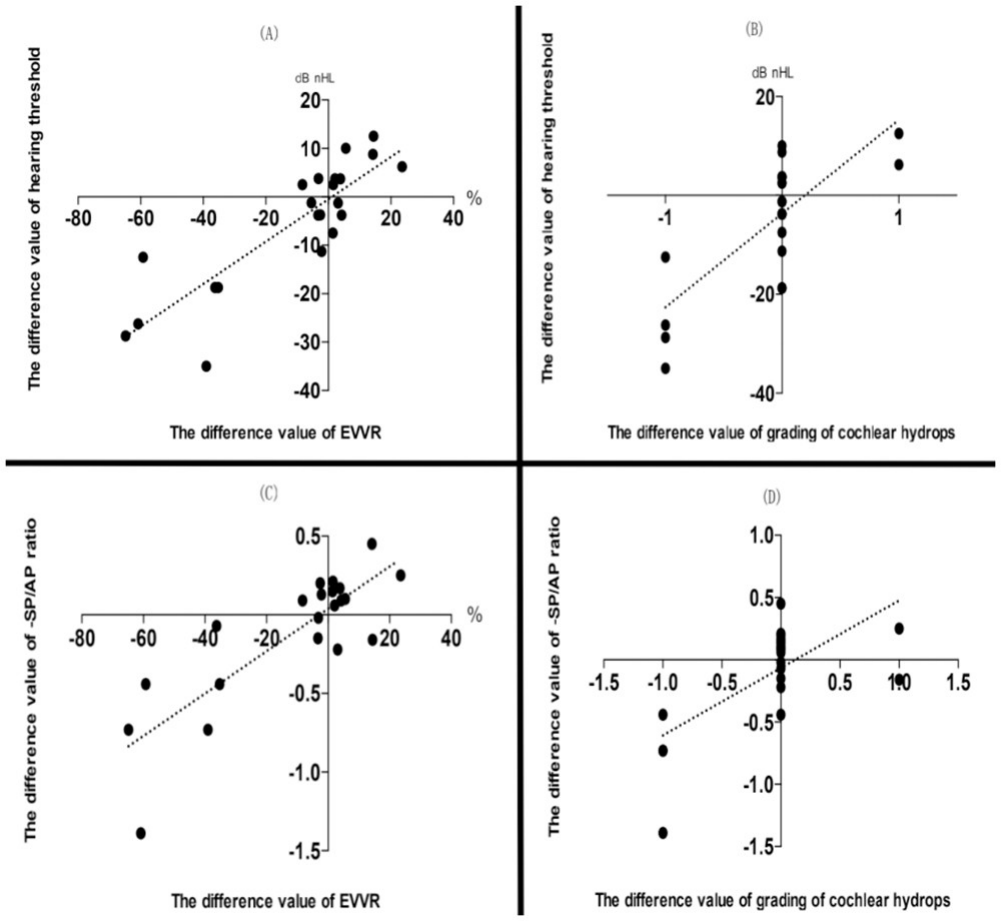
Correlation between the difference value of the hearing threshold, -SP/AP ratio of EcochG, EVVR and grading of cochlear hydrops prior to and following surgery. (A)The difference value of the hearing threshold had a significant positive correlation with the difference value of the EVVR (P = 0.000, r = 0.739). (B) A significant positive correlation was observed between the difference value of the hearing threshold and grading of cochlear hydrops (P = 0.000, r = 0.562). (C) The difference value of the -SP/AP ratio was significantly correlated with the difference value of the EVVR (P = 0.000, r = 0.690). (D) A significant positive correlation was identified between the difference value of the -SP/AP ratio and grading of cochlear hydrops (P = 0.001, r = 0.467). -SP/AP, summating potential/action potential ratio; EVVR, endolymph to vestibule-volume; EcochG, electrocochleography.

**Fig 7:**
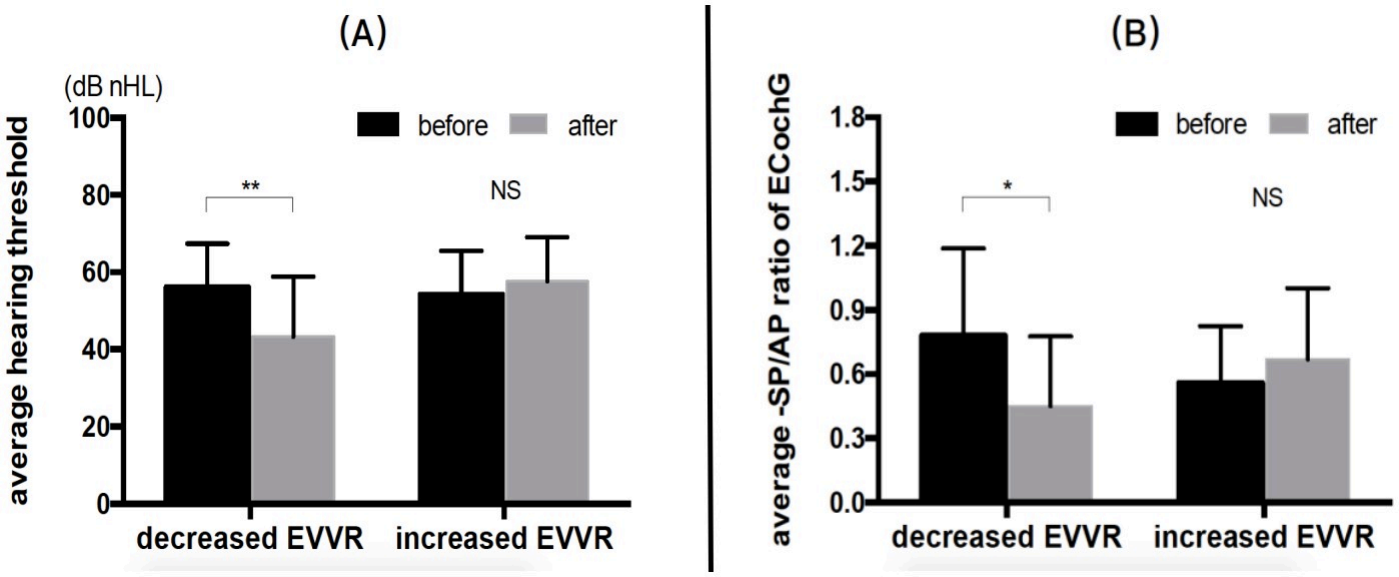
Changes in the average hearing threshold and -SP/AP ratio of EcochG. Among patients with a decreased EVVR, the (A) average hearing threshold (43.27±15.60) and (B) -SP/AP ratio (0.45±0.32) decreased significantly following surgery, as compared with the value prior to surgery (56.06±11.33, P = 0.005 and 0.78±0.41, P = 0.049, respectively). Among patients with an increased EVVR, the (A) average hearing threshold (57.64±11.40) and (B) -SP/AP ratio (0.67±0.33) tended to increase following EDB, as compared with the value prior to it (54.16±11.40 and 0.56±0.26, respectively), but there was no significant difference between the two groups (P = 0.115 and P = 0.105, respectively). -SP/AP, summating potential/action potential ratio; EcochG, electrocochleography; EVVR, endolymph to vestibule-volume; EDB, endolymphatic duct blockage; **P < 0.01, paired t-test; *P < 0.05, paired t-test; NS, not significant, paired t-test.

As shown in Table 1, the postoperative average hearing threshold in 22 patients was 49.80±15.39, which was not significantly different (P = 0.067) from that prior to surgery (55.20±11.13). However, the difference value of the hearing threshold had a significant positive correlation with the difference value of the EVVR (P = 0.000, r = 0.739) (Fig 6A) and grading of cochlear hydrops (P = 0.000, r = 0.562) (Fig 6B) between prior to and following surgery. Among patients with a decreased EVVR, the average hearing threshold (43.27±15.60) decreased significantly following surgery, as compared to that prior to surgery (56.06±11.33) (P = 0.005) (Fig 7A). Six out of seven patients with a hearing improvement (≥10-dB decline) exhibited downgrading of their hydrops in the cochlea and/or vestibule (patient nos. 4, 5, 10, 12, 14 and 16) (Table 1). Yet, among patients with an increased EVVR, the average hearing threshold (57.64±11.40) tended to increase following EDB, as compared with that prior to it (54.16±11.40), but there was no significant difference between the two groups (P = 0.115) (Fig 7A). One of two patients with hearing deterioration (≥10-dB elevation) showed upgrading of her hydrops in both cochlea and vestibule (patient no. 8) (Table 1).

### Modulation of -SP/AP ratio of EcochG following dynamic changes of hydrops

Table 1 shows 73% (16/22) of patients had a positive EcochG (-SP/AP ratio >40%) prior to surgery and 55% (12/22) of patients had a positive EcochG following surgery; there was no significant difference between the two groups (P = 0.190). The mean preoperative SP/AP ratio of ECochG on the affected side was 0.67±0.36, and the postoperative ratio 0.55±0.34, with no significant difference identified (P = 0.217).

However, the difference value of the -SP/AP ratio had a significant positive correlation with the difference value of the EVVR (P = 0.000, r = 0.690) (Fig 6C) and grading of cochlear hydrops (P = 0.001, r = 0.467) (Fig 6D) between prior to and following surgery. Among patients with a decreased EVVR, the average -SP/AP ratio (0.45±0.32) decreased significantly following surgery, as compared with that prior to surgery (0.78±0.41) (P = 0.049) (Fig 7B), and 4/5 patients with a negative conversion of EcochG exhibited downgrading of their hydrops in both cochlea and vestibule (patient nos. 4, 5, 12 and 14) (Table 1). Among patients with an increased EVVR, the average -SP/AP ratio (0.67±0.33) tended to increase following EDB, as compared with that prior to surgery (0.56 ±0.26), but there was no significant difference between the two groups (P = 0.105) (Fig 7B).

## Discussion

In vivo visualization of hydrops with Gd-MRI is no longer limited to only showing evidence of the hydrops for the diagnosis of MD. Changes in EH may be used to objectively evaluate the effects of various treatments for patients with MD [19,20]. High-quality imaging is critical for evaluating the dynamics of hydrops, although promising results have been reported using heavily T2-weighted 3D FLAIR sequence following i.v. administration, which has the advantage of not being an off-label use of Gd [21,22]. Yet, the image analysis protocols based on image calculations were too complicated and time-consuming to use for routine diagnostic procedures [23–25]. However, it was difficult to obtain serial MRI scans by this method for evaluating the dynamic changes of EH of “after treatment” and “before treatment” groups. In the present study, the use of IT-Gd + IV-Gd MRI not only improved the effectiveness of imaging and evaluation techniques for EH [26], but also prevented the failure of IT-Gd imaging for ~10% of cases, which would have been caused by an insufficient Gd concentration with anatomic barriers to the round window, such as adhesions, bone dust blockage or thickened round window, when the IT-Gd method alone was used [27,28]. In fact, none of the subjects showed no or diagnostically insufficient inner ear contrast in this study. As shown in Figs 4 and 5, IT-Gd + IV-Gd MRI showed the dynamic changes of EH with serial MRI scans in patient no. 14 prior to surgery, and 2 weeks, 10 months and 16 months after surgery. A discernable reversal of EH accompanied by a hearing improvement and a negative conversion of EcochG could be detected 16 months after surgery.

Although the pathology of MD is well established to be EH. However, the mechanism underlying deafness of MD or idiopathic endolymphatic hydrops is still unknown. The most popular assumption is that the low-tone hearing loss is caused by the displacement of the basilar membrane (BM) from its resting position and the uncoupling of the outer hair cell stereocilia from the tectorial membrane due to hydrops [29–31]; this is known as the mechanical hypothesis. In this theory, the hearing loss in MD is purely due to increased mechanical pressure in the cochlear duct [32]. Yet, it remains unclear whether the hearing loss in MD is caused by hydrops alone and whether the hearing function can be modulated by changes in the hydrops. A dehydration test via an osmotic diuretic to abruptly diminish hydrops is usually used to observe temporary reversal of hearing loss in MD [33–37]. Diuretic-induced endolymphatic dehydration has been radiologically observed in humans [38], with considerable evidence confirming that glycerol-induced endolymphatic volume load reduction occurs in animals [39–42]. In addition, the reduction of endolymphatic fluids resulting in hearing improvement has been described upon surgical observation; in cases where there was a flow of endolymph following drainage, an improvement in hearing at the low-pitched tone was observed, while in cases where there was no flow of endolymph, no improvement in hearing occurred [43]. The aforementioned findings suggested a likelihood that the hearing loss may be recoverable if the underlying hydrops can be corrected.

Based on a mechanical theory about hearing loss in MD, the low-tone sensorineural hearing loss in MD is caused by the mechanical impact of high endolymphatic pressure [44], which deflects the BM towards the scala tympani and becomes the basis for elevations in the summating potential detected by ECochG. In the present study, among patients with decreased EVVR, the average hearing threshold and -SP/AP ratio decreased significantly; among patients with an increased EVVR, the average hearing threshold and -SP/AP ratio tended to increase following surgery, as compared with the value prior to surgery (Fig 7). These results showed a causative correlation between a disordered hearing function and EH [45], in which the reduction of EH is likely to decrease the direct mechanical impact of high endolymphatic pressure and reverse the displacement of the BM, leading to an improvement in hearing. On the contrary, the increased of endolymphatic volume may give rise to an increase in the displacement of the BM and result in hearing deterioration. Although no significant differences were observed between the postoperative and preoperative average hearing threshold and -SP/AP ratio of EcochG in 22 patients that had undergone EDB, the difference value of the hearing threshold and -SP/AP ratio were significantly positively correlated with the difference value of the EVVR and grading of cochlear hydrops between prior to and following surgery, respectively (Fig 6), which could further strengthen the relationship between the dynamics of EH and changes in the hearing function. In addition, 6/7 patients with a hearing improvement (≥10-dB decline) and 4/5 patients with a negative conversion of EcochG exhibited downgrading of their hydrops in the cochlea and/or vestibule(Table 1), suggesting a downgrading of the cochlear and/or vestibular hydrops accompanied by the downregulation of the hearing threshold and -SP/AP ratio of EcochG. One out of two patients with a hearing deterioration (≥10-dB elevation) exhibited upgrading of the hydrops in both cochlea and vestibule, suggesting that the upgrading of cochlear and vestibular hydrops tended to upregulate the hearing threshold and -SP/AP ratio of EcochG.

Recently, Ito et al reported a positive correlation between the control of vertigo and the decrease in the volume of EH, and no correlation between changes in the hearing function and the volume of EH following sac drainage surgery [46,47]. Higashi-Shingai et al also described that the presence of vestibular EH and the frequency of vertigo attacks significantly decreased following surgery, but no significant improvement in the presence of cochlear EH or the SP/AP ratio of ECochG was observed [48]. The present results showed an obvious difference in the relationship between hearing and -SP/AP ratio, and dynamic changes in the hydrops, as compared with the results of former studies, which could be explained by the varying effects of different methods. In our experience, the reduction in EH could be achieved in certain patients with MD following sac drainage surgery immediately, but there was no improvement in hearing in the majority of these patients who were confirmed with the reversal of hydrops (data not shown), which was consistent with the results of former studies [46–48]. However, in the group of patients who underwent EDB, the reduction of EH could not be observed immediately following surgery (Fig 4B) and the hydrops remained unchanged 2 weeks after surgery. The reversal of EH could be detected only months after surgery (Fig 4C and 4D), which was more likely to be in accordance with the physiological recovery in the homeostasis of the endolymph. Although the exact role of the EDB procedure on EH remains unknown, the mechanism of action of EDB in MD treatment is expected to be different from that of the traditional ES shunting/decompression procedures [18]. Nevertheless, discussing the difference of these ES surgical procedures was beyond the scope of this study, which focused on evaluating the morphologic-functional correlation between the dynamic change of endolymphatic fluids and hearing function in MD patients following EDB. It was found that there is a great likelihood that hearing loss in MD is caused by EH, and the downgrading or upgrading of EH morphologically holds great potential for modulating hearing function.

The limitation of the present study is the small number of patients. Although fluctuation in the composition and volume of the endolymph is considered to contribute to the fluctuating nature of the symptoms experienced by sufferers of Meniere’s. On average, MD stabilizes with no further vestibular attacks by about 8 years after the onset of symptoms; however, hearing loss typically becomes permanent as MD progresses [49]. Nevertheless, by comparing the proportions of the EH ratio of “after treatment” and “before treatment” groups, we could understand the extent to which EH could be reduced to improve hearing. This could facilitate future progress in the field of treatment for MD, especially for maintenance or improvement of hearing in MD.

In conclusion, in the present study, it was first reported that the dynamic change of EH visualized by Gd-MRI was likely to modulate hearing function in 22 MD patients following EDB, highlighting the possibility that the downgrading of cochlear and/or vestibular hydrops could downregulate the hearing threshold and -SP/AP ratio, whereas the upgrading of cochlear and/or vestibular hydrops tended to upregulate the hearing threshold and -SP/AP ratio, suggesting that the hearing loss in MD is likely to be caused by hydrops and this disordered hearing function can be modulated by changes in the hydrops.

## Supporting information

S1 File STROBE statement—Checklist of items that should be included in reports of observational studies. (DOCX)
